# A 2-year-old girl with chronic crackles after respiratory syncytial virus infection: a case report

**DOI:** 10.1186/s13256-018-1797-6

**Published:** 2018-09-12

**Authors:** Katarzyna Woicka-Kolejwa, Henryk Mazurek, Iwona Stelmach

**Affiliations:** 1 Department of Pediatrics and Allergy, Medical University of Lodz, Lodz, Poland; 2 Department of Pneumonology and Cystic Fibrosis, Institute of Tuberculosis and Lung Disorders, Rabka–Zdrój, Poland; 3 Copernicus Memorial Hospital, Korczak Paediatric Center, Piłsudskiego 71 Str, 90-329 Lodz, Poland

**Keywords:** Infections, Pneumonia, RSV, Viral, Interstitial lung disease (ILD)

## Abstract

**Background:**

Respiratory syncytial virus is the most common cause of lower respiratory tract infections in infants and young children. While the majority of infants display only mild upper respiratory tract infection or occasionally otitis media, around one-third will develop an infection of the lower respiratory tract, usually bronchiolitis. There is now convincing evidence from a number of cohorts that respiratory syncytial virus is a significant, independent risk factor for later wheezing, at least within the first decade of life. The wide variation in response to respiratory syncytial virus infection suggests that susceptibility and disease are influenced by multiple host-intrinsic factors.

**Case presentation:**

A 2-year-old white girl presented to our Pediatric Allergy Clinic with recurrent crackles in addition to cough, fevers, and labored breathing since her first respiratory syncytial virus infection at the age of 7 months. She had been under the care of pulmonologists, who suspected childhood interstitial lung disease. She was hospitalized eight times due to exacerbation of symptoms and prescribed systemic and inhaled steroids, short-acting β2-mimetics, and antileukotriene. There was no short-term clinical improvement at that time between hospitalizations.

During her hospital stay at the Pneumonology and Cystic Fibrosis Department in Rabka a bronchoscopy with bronchoalveolar lavage was performed. Laboratory bacteriological tests found high colony count of *Moraxella catarrhalis* (β-lactamase positive), sensitive to amoxicillin-clavulanate, in bronchial secretions and swabs from her nose. After this, infections were treated with antibiotics; she remained in good condition without symptoms. Crackles and wheezing recurred only during symptoms of infections. Therefore, we hypothesize that respiratory syncytial virus infection at an early age might cause severe damage of the lung epithelium and prolonged clinical symptoms, mainly crackles and wheezing, each time the child has a respiratory infection.

**Conclusions:**

This case illustrates the importance of respiratory syncytial virus infection in an immunocompetent child. Pediatricians need to have a high index of suspicion and knowledge of recurrent symptoms associated with severe damage of the lung epithelium to establish the correct diagnosis.

## Background

Respiratory syncytial virus (RSV) is the most common cause of lower respiratory tract infections (LRTIs) in infants and young children [1]. By the age of 2 years, over 80% of children have experienced at least one RSV infection, two-thirds of these occurring in the first year of life. Strong epidemiologic evidence suggests that early-life infections with this virus predispose to chronic respiratory dysfunction and even asthma, possibly related to persistence of the virus itself or to its effects on lung development [2]. While the majority of infants display only mild upper respiratory tract infection (URTI) or occasionally otitis media, around one-third will develop an LRTI, usually bronchiolitis. This is caused by an infiltration of inflammatory cells into the air spaces, mucus hyper-production, shedding of necrotic airway epithelial cells, and edema of the airway wall. These processes lead to a narrowing of the airway lumen, airflow obstruction, overinflation, and impaired gas exchange. In more severe RSV disease, crackles and wheeze occur with labored breathing, tachypnea, and hypoxia; a small percentage of cases require intensive care and may result in death [3]. There is now convincing evidence from a number of cohorts that RSV is a significant, independent risk factor for later wheezing, at least within the first decade of life. The wide variation in response to RSV infection suggests that susceptibility and disease are influenced by multiple host-intrinsic factors.

## Case presentation

We present a case of 2-year-old white girl with chronic crackles admitted to our Pediatric and Allergy Clinic. The pregnancy was unremarkable and after birth the child was healthy until the seventh month of life, when she developed RSV infection. From then on she had a LRTI every month treated with antibiotics, mainly macrolides for presumed bacterial pneumonia; symptoms persisted daily. She had been under the care of pulmonologists from a different department, who suspected childhood interstitial lung disease (chILD) and prescribed systemic and inhaled steroids, short-acting β2-mimetics, and antileukotriene. This treatment, however, did not lead to any clinical improvement; symptoms of crackles were present at all times. She was hospitalized eight times due to exacerbation of symptoms such as dyspnea, cough, and persistent crackles during physical examination. At the age of 11 months she had high resolution computed tomography (HRCT) which revealed lung areas of uneven aeration in the middle lobe of her right lung and small areas of densities which indicated postinflammatory changes. Due to suspected *Pneumocystis jirovecii* (*carinii*) infection, she was unsuccessfully treated with sulfamethoxazole and trimethoprim.

**Fig. 1:**
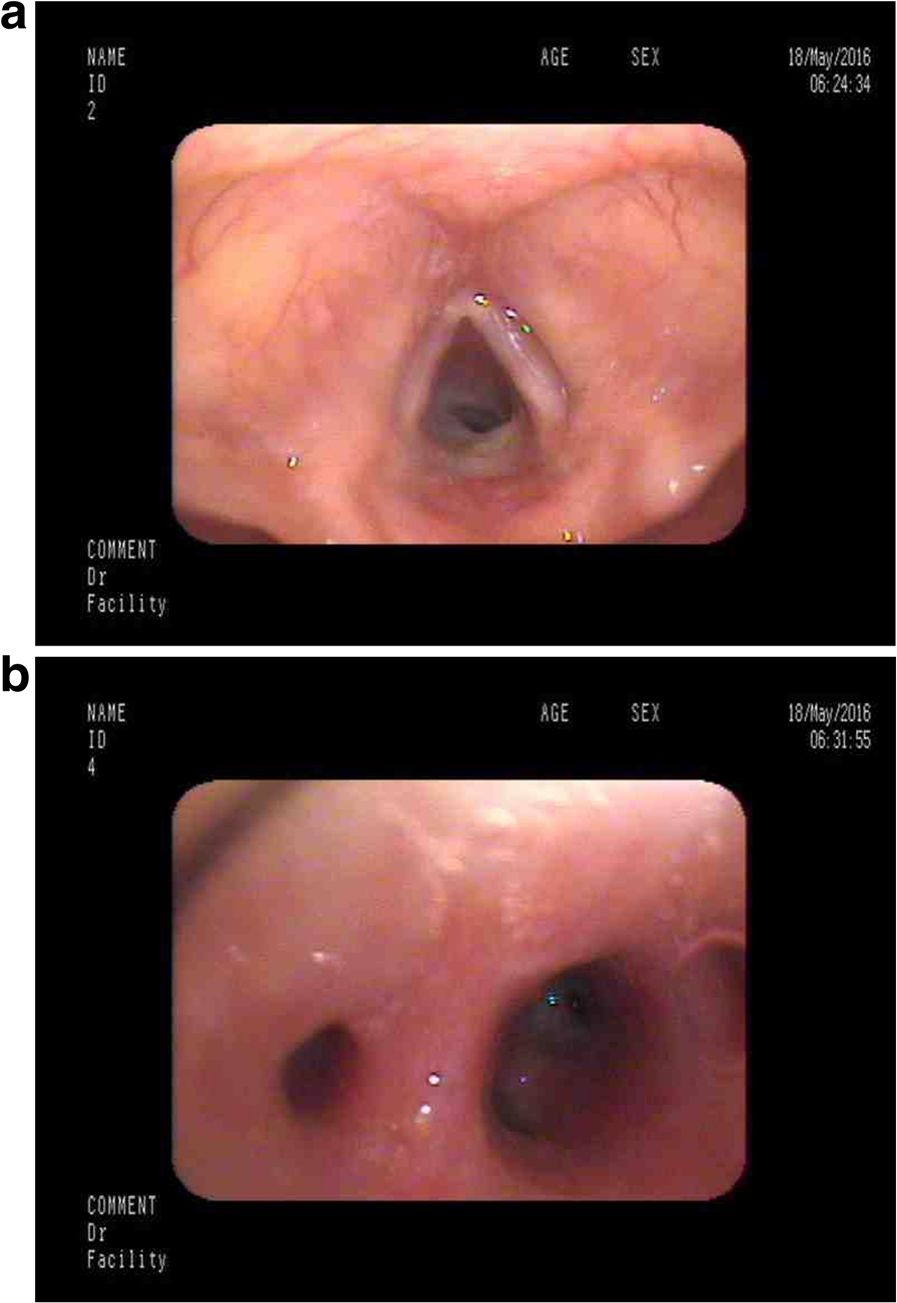
**a** Bronchoscopy image – mucosal edema of the larynx. **b** Bronchoscopy image – purulent mucus in bronchi

She was admitted to our clinic at 23 months of age with intense cough, dyspnea, and chronic crackles. A chest X-ray showed areas of density due to parenchymal and interstitial inflammatory changes. Autoimmune disease and atypical inflammatory infections (*Mycoplasma pneumoniae, Chlamydia pneumoniae,* and *Bordetella pertussis*) were excluded by use of a multiplex assay; immunodeficiency was also excluded. An echocardiogram revealed no abnormalities. Next, she was referred to the Pneumonology and Cystic Fibrosis Department in Rabka for bronchoscopy with bronchoalveolar lavage (BAL). The result showed: copious purulent secretions in her lower throat; mucosal edema of the larynx (Fig. 1a), trachea, and bronchial tree; and retention of the purulent mucus in bronchi (Fig. 1b) with normal movement of bronchial cilia (high frequency video microscopy). Microbiological testing with growth on blood/chocolate agar isolated high colony count of *Moraxella catarrhalis* in the BAL fluid. It was beta-lactamase producer sensitive to amoxicillin-clavulanate. The BAL also showed epithelial cells, macrophages, and neutrophils under high power field. She was administered amoxicillin-clavulanate for 14 days with good clinical improvement in respiratory rate, labored breathing, and cough and she was discharged. She was observed for 2 months after discharge from the hospital and showed no signs of recurrence. Then, she had a few more respiratory tract infections (usually every other month) treated with antibiotics (crackles were present at each time during infection); between infections she remained healthy, without any crackles or wheezing.

## Discussion

This case illustrates RSV pneumonia in an immunocompetent child. The chronic character of symptoms, persistent crackles, and the lack of improvement after treatment with systemic and inhaled steroids, short-acting β2-mimetics, and antileukotriene at first suggested interstitial lung disease. Interstitial lung disease is a rare condition in childhood, with an estimated prevalence of 0.36/100,000; therefore, pediatricians may be less familiar with interstitial lung disease [4]. To diagnose chILD at least three of four criteria should be fulfilled [5, 6]. Our patient does not meet the criteria for chILD as the symptoms were not present since birth and there was no hypoxia. In addition, computed tomography (CT) findings did not show signs such as septal thickening, ground glass opacification, geographic hyperlucency or mosaic attenuation, lung cysts or nodules, and consolidation suggestive of chILD. What is more, CT scans did not suggest bronchiolitis obliterans, neuroendocrine cell hyperplasia of infancy, or other airway pathology. Later, a bronchoscopy with BAL revealed *M. catarrhalis* infection. *M. catarrhalis* is a common commensal as well as a pathogen of the human respiratory tract. In our patient, after this infection was treated with an antibiotic, she remained in good condition without symptoms; crackles and wheezing recurred only during infection. Pulmonary function testing was not done due to her young age. We hypothesize that RSV infection at an early age might cause severe damage of the lung epithelium and prolonged clinical symptoms, mainly crackles and wheezing, each time the child has respiratory infection. In this case, the main prophylactic of recurrent clinical symptoms is to avoid exogenous infections by additional prophylactic vaccination. Symptoms resolution after treatment with antibiotics of this and later infections suggest that initial RSV infection in early childhood has a role.

## Conclusions

This case illustrates the importance of RSV infection in an immunocompetent child. Pediatricians need to have a high index of suspicion and knowledge of symptoms associated with severe damage of the lung epithelium to establish the correct diagnosis.

## Abbreviations

BAL: Bronchoalveolar lavage
chILD: Childhood interstitial lung disease
CT: Computed tomography
HRCT: High resolution computed tomography
LRTI: Lower respiratory tract infection
RSV: Respiratory syncytial virus
URTI: Upper respiratory tract infection
